# Liquid crystalline 2D borophene oxide for inorganic optical devices

**DOI:** 10.1038/s41467-022-28625-w

**Published:** 2022-02-24

**Authors:** Tetsuya Kambe, Shotaro Imaoka, Misa Shimizu, Reina Hosono, Dongwan Yan, Hinayo Taya, Masahiro Katakura, Hirona Nakamura, Shoichi Kubo, Atsushi Shishido, Kimihisa Yamamoto

**Affiliations:** 1 Laboratory for Chemistry and Life Science, Tokyo Institute of Technology, Yokohama, 226-8503 Japan; 2 JST-ERATO, Tokyo Institute of Technology, Yokohama, 226-8503 Japan; 3 Kanagawa Institute of Industrial Science and Technology (KISTEC), Tokyo Institute of Technology, Yokohama, 226-8503 Japan

**Keywords:** Polymer chemistry, Polymers

## Abstract

Borophene has been recently proposed as a next-generation two-dimensional material with promising electronic and optical properties. However, its instability has thus far limited its large-scale applications. Here, we investigate a liquid-state borophene analogue with an ordered layer structure derived from two-dimensional borophene oxide. The material structure, phase transition features and basic properties are revealed by using X-ray analysis, optical and electron microscopy, and thermal characterization. The obtained liquid crystal exhibits high thermal stability at temperatures up to 350 °C and an optical switching behaviour driven by a low voltage of 1 V.

## Abstract (web summary)

Borophene exhibits attractive electronic and optical properties, but its instability has so far limited its applications. Here, the authors report the synthesis of a liquid-state borophene analogue showing a good thermal stability up to 350 °C and an electrically-controlled optical switching behaviour.

## Introduction

Borophene^1–4^ is a boron-based two-dimensional (2D) atomic layered material. Such single atomic layers and their analogues, represented by borophene or graphene, are promising materials for electronic devices because of their Dirac electronic behavior. In addition, borophene has the potential to exhibit high flexibility^5,6^ due to its boron 2D network structure. The high flexibility of borophene is advantageous for the generation of a liquid state at low temperatures. However, the phase transition of the original borophene is severely limited by its poor stability, as it survives only on a substrate^1,3^. In contrast, borophene oxide, which has been previously predicted^7,8^ and recently investigated^9^, can resolve the stability problem of borophene by improving the stability of the hexagonal boron network. In addition, the borophene oxide layers (BoLs) reported by Kambe et al. contain intercalated potassium cations, which can weaken the interactions of interlayers^9^.

2D networked structures can retain a coplanar arrangement of atoms even if the crystalline structure collapses, unlike the case of small molecules. Therefore, it is advantageous to form a liquid crystalline state since the in-plane arrangement of atoms is maintained in the liquid state. Such liquid crystals derived from 2D structures are new materials that are attracting much attention for the development of optoelectronics and photonics accompanied by applications of films, fibers, membranes and optoelectronic devices^10^. For example, clay minerals^11^, graphene^12^, and graphene oxide^13^ have been used as lyotropic liquid crystals (LCs)^14^ prepared in solutions. However, the precipitation effect induced by changes in concentration makes their use as functional materials difficult. Here, we demonstrate a borophene oxide analogue as a fully inorganic liquid with a layered structure and extraordinary thermal stability, suggesting that it is a candidate for use in optical devices to compensate for the lack of suitable common organic liquid crystals^15–23^. Although a liquid crystal device using graphene oxide has been reported, it was a lyotropic liquid crystal depending on the solution concentration^24^. Therefore, the previously reported material is different from the liquid borophene without any solvents shown in this work. As optical materials, atomically thin 2D sheets are also attracting much attention due to their 2D-type plasmons^25–28^. In particular, metallic borophenes with a Dirac-type band structure are expected to exhibit excellent electronic and optical properties, including low-loss and highly confined broadband plasmons^29–32^.

## Results

### Preparation of liquid borophene oxide

**Fig. 1:**
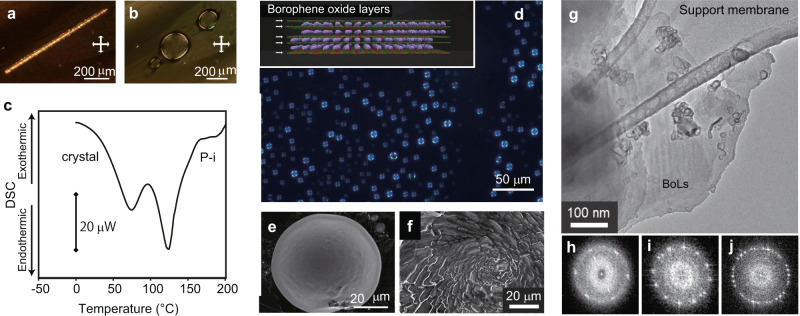
Change from the crystal phase to the layered liquid phase. Polarized optical microscopy images of a crystal of borophene oxide layers (BoL-C) (**a**) and liquid crystals of borophene oxide layers (BoL-LC) (**b**) under crossed polarizers (white double arrows). **c** Differential scanning calorimetry (DSC) heating curve corresponding to the change from the crystal to BoL-LC phase (P-i) in a glass capillary. **d** Optical microscopic image of BoL-LC droplets. The image was obtained under crossed polarizers with a sensitive tint plate. The inset of **d** shows a chemical structure of the prepared BoLs. White arrows indicate the borophene oxide atomic layers. The purple spheres represent potassium cations. **e** A scanning electron microscope (SEM) image for a drop of BoL-LC. **f** Close-up of the central position of the liquid crystal after solidification by air oxidation process. **g** A transmission electron microscope (TEM) image of BoL-LC on a microgrid. **h**–**j** Electron beam diffraction patterns correspond to single, double, and several layers, respectively.

BoLs, which possess boron hexagonal atomic layers with potassium cations, were fabricated according to a previously reported method as a crystal of borophene oxide layers (BoL-C)^9^. BoL-C was changed to a liquid crystal of borophene oxide layers (BoL-LC) as a result of heating (Fig. 1a, b) in the temperature range of 105–120 °C. The change from BoL-C to BoL-LC was monitored by differential scanning calorimetry (DSC). The DSC curve for a BoL-C sample in a glass capillary under vacuum showed two endothermic peaks at ~70 and 120 °C (Fig. 1c). Polarized optical microscopy using crossed polarizers showed a bright area at the periphery of every liquid drop in conjunction with four black points at the crossed position, suggesting a spherulite structure generated by an ordered lamellar-like pattern (Fig. 1d, Supplementary Fig. 1, Supplementary Movie 1). In the case of spherulite structures derived from nanosheets, two structural types are possible according to the stacking directions of the sheets. One type is the radially stacked structure, in which the normals of the sheets match the tangents of the drop; that is, the edges of the sheets are oriented towards the center and outside of the drop (Supplementary Fig. 2a). This form gives a strong interference color at the center area of the drop. For the other structural type, the normals of the boron sheets are oriented towards the center of the drop. In this case, the stacked sheets are arrayed parallel to the surface of the drop with a slightly curved form (Supplementary Fig. 2b). In addition, the 2D layers at the periphery are stacked in a more orderly fashion than those in the center area. In the case of BoL-LC, the observed interference color of the sample was bright at the peripheral area, suggesting the latter structure type (Supplementary Fig. 2b). This spherulite structure was also confirmed by scanning electron microscopy (SEM) observation after solidification induced by an oxidation treatment (Fig. 1e, f). The self-organization ability of this spherulite liquid crystal was clearly demonstrated by cutting the drop (Supplementary Movie 2). Both a thin-layer structure and a hexagonal network were observed in BoL-LC by transmission electron microscopy (TEM) and electron-beam diffraction analysis (Fig. 1g–j). We observed diffraction patterns generated by single, double, and several hexagonal layers by changing the measurement positions (Fig. 1h–j, Supplementary Fig. 3). The *d*-spacing resulting from these measurements (*d* = 0.20 nm) is nearly equal to the (210) in-plane distance (*d* = 0.199 nm), which was obtained by crystal-structure analysis of BoL-C^9^.

**Fig. 2:**
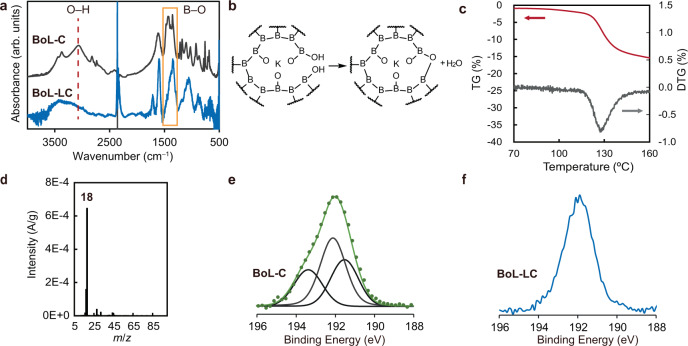
Chemical change from BoL-C to BoL-LC. **a** Infrared (IR) spectrum change from BoL-C to BoL-LC. The peak of O–H stretching (red dashed line) was disappeared and the peak shape of the B–O stretching (orange solid line) was changed. Solid line around 2400 cm^−1^ is derived from CO_2_ gas in the air. **b** Considerable chemical structure change. Dehydration process from hydroxyl groups on boron (B-OH) to form oxygen bridging structure occurs at the edge parts. The distorted edge structure prevents the 2D layers from crystallization. This change assists the formation of the liquid crystal phase. **c** Thermogravimetry (TG) and differential thermogravimetry (DTG) spectra of the reaction from BoL-C to BoL-LC. **d** Mass spectra (MS) at 130 °C of TG-MS measurement. The peak corresponding to H_2_O was observed at the position of *m*/*z* = 18. *m* stands for mass and *z* stands for charge number of ions. **e**, **f** XPS spectra of BoL-C and BoL-LC at B 1 *s* region, respectively.

According to the DSC measurements, the addition of inert gas to the capillary shifted the peak at 70 °C (Fig. 1c) to 110 °C (Supplementary Fig. 4a), suggesting that this peak resulted from the vaporization of solvent on the crystal surfaces. In contrast, the peak at 120 °C maintained its position (Supplementary Fig. 4a). The thermogravimetric analysis (TGA) and differential thermogravimetry (DTG) data both show a decrease in mass corresponding to the DSC peaks (Supplementary Fig. 4b). By comparison with data acquired from B(OH)_3_, the peak at 120 °C in Fig. 1c was assigned to the dehydration of B-OH groups in BoL-C (Supplementary Fig. 4b, c). The dehydration process of B-OH groups is supported by the variations in the Fourier transform infrared (FT-IR) spectra showing the disappearance of the O–H stretching peak and changes in the B–O stretching peaks (Fig. 2a). These data suggest that dehydration at the edges of layers or defects in BoL-C weakened the interactions between layers (Fig. 2b), producing the liquid phase. The removal of H_2_O was confirmed by thermogravimetry-mass spectrometry (TG-MS). Chemical species with a molecular weight of 18 were detected by MS when the sample lost mass during heating (Fig. 2c, d). Chemical changes associated with B-OH at the edge regions were also observed during X-ray photoelectron spectroscopy (XPS) analysis. Specifically, the peak at 193.4 eV in the spectrum of BoL-C (Fig. 2e) was not present in the spectrum of BoL-LC (Fig. 2f).

### Phase transition feature of liquid borophene oxide

**Fig. 3:**
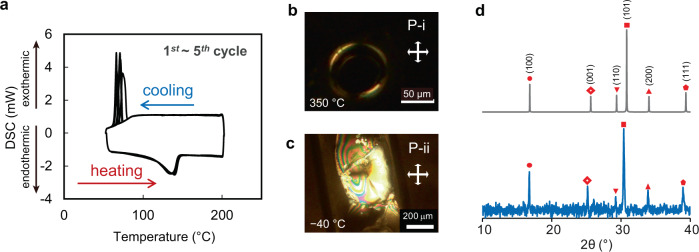
Thermal properties of BoL-LC. **a** DSC curves corresponding to cooling and heating of BoL-LC. BoL-LC transitions from the P-i to the P-ii phase at ~100 °C during cooling and from the P-ii to the P-i phase at approx. 150 °C during heating. The P-i (**b**) and P-ii (**c**) phases in a glass capillary observed by polarized optical microscopy under crossed polarizers (white double arrows) at room temperature. **d** X-ray diffraction (XRD) peaks of the sample at room temperature (blue). The spectra in gray show peaks calculated from the obtained single-crystal data of BoL-C.

BoL-LC exhibited a phase transition (P-ii/P-i) at ~100 °C during both the cooling and heating processes. This transition was evident from the exothermic peak that appeared in the DSC cooling curve coming from the P-i phase (Fig. 3a). DSC analysis of the P-ii phase also showed a single, broad endothermic peak at approx. 150 °C. The difference in the transition temperatures during heating and cooling indicates a supercooling phenomenon in the P-i phase. The phase transition was also clearly identified from polarized optical microscopy images (Fig. 3b, c, Supplementary Fig. 5a). Although the P-ii phase was different from BoL-C, an ordered structure was observed throughout the sample (Fig. 3c). The P-ii phase was characterized by X-ray diffraction (XRD) analysis at room temperature. The (001), (101), and (111) peaks in the pattern of the P-ii phase were shifted compared with those of BoL-C (Fig. 3d). Each of these shifted peaks was attributed to diffraction planes in the *c*-axis direction. In contrast, the (100) and (110) peaks corresponding to planes not in the *c*-axis direction were unchanged, indicating that the *c*-axis of BoL-C expanded slightly when BoL-LC was changed. We consider that this expansion is due to the chemical change in defective B-OH structures via the dehydration process, as shown in Fig. 2. The liquidity of the P-ii phase was also demonstrated in a sample prepared by rapid cooling of the P-i phase from 200 to 20 °C. In this case, the gradual melting of the P-ii phase at 20 °C was observed by polarized optical microscopy (Supplementary Fig. 5b).

Both phases were found to exhibit high thermal stability over a wide temperature range. Polarized optical microscopy images of the ordered phases showed no changes over the range from −40 to 350 °C other than the phase transition between P-i and P-ii (Fig. 3b, c). The P-i phase was stable at 350 °C (above this temperature, the phase decomposed; see Supplementary Figs. 6 and 7). In addition, DSC curves acquired at −50 °C for P-ii did not show any phase changes (Supplementary Fig. 8).

### Anisotropic properties of BoL-C and BoL-LC

Anisotropic properties of BoL-C and BoL-LC samples were observed by polarized optical microscopy with crossed Nicols by changing the angles of the samples (Supplementary Fig. 9). For BoL-C, bright and dark conversions were clearly observed every 45°, indicating that the crystal has different refractive indices in the long and short axes (Supplementary Fig. 9a). The structure of BoL-C was shown in a previous report, and the column-shaped crystals were revealed to be formed by the lamination of borophene sheets^9^. The results of the optical measurements strongly reflect the structure. In the case of BoL-LC, the optical patterns changed according to the orientation of the borophene sheets. In the P-ii phase, interference colors were observed over the entire area of the sample. Especially in the central part of the images, bright and dark conversions were clearly observed every 45° (Supplementary Fig. 9b). In contrast, the P-i phase exhibited 4 dark positions at the periphery of the droplet. The crossed optical pattern was maintained even when the angles changed. This result demonstrated the spherulite orientation of the borophene sheets (Supplementary Fig. 9c). Images taken with a polarized optical microscope with a tint plate inserted are shown in Supplementary Fig. 10. Additive and subtractive colors were clearly observed as the stage was rotated, showing the uniaxial orientation of the sample. The retardation (*R*) values of the P-ii phase at positions A (thickness: 25 μm) and B (thickness: 10 μm) shown in Supplementary Fig. 10 were measured using a Berek compensator. The observed values of *R* were 486 nm and 241 nm, respectively, and the birefringence (Δ*n*) values were 0.019 (A) and 0.024 (B).

### Optical properties for device applications

**Fig. 4:**
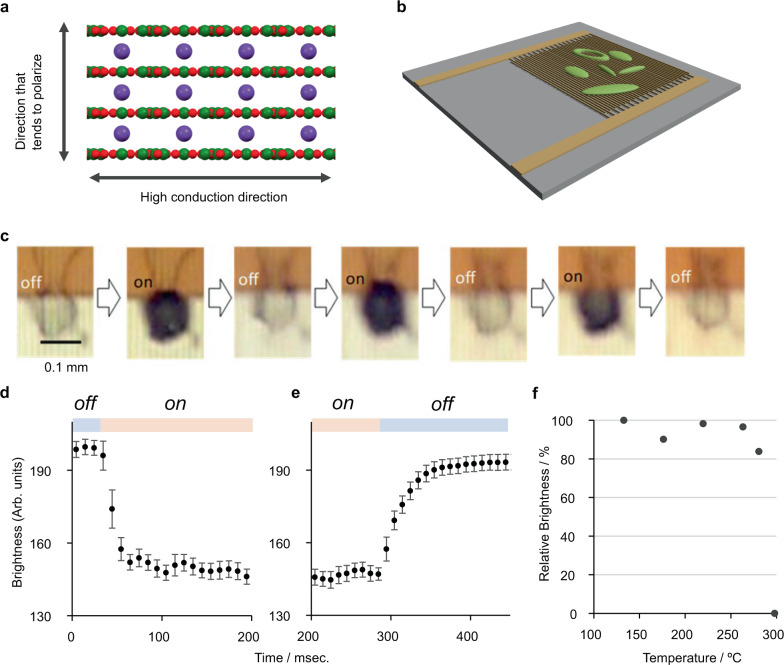
BoL-LC inorganic optical device. **a** Schematic of the conceivable dynamic scattering phenomenon by BoL-LC. Spheres in the structure represent the constituent elements (green: boron, red: oxygen, purple: potassium). **b** Diagram of a BoL-LC device fabricated using a comb electrode. **c** On/off optical switching induced by an applied voltage (1 V). Response speed of the device; **d**, **e** correspond to the changes of the on and off states, respectively. The error bars in **d**, **e** stand for the standard deviation of ten observed values. **f** Temperature dependency of the repetition feature in the BoL-LC device.

BoL-LC is expected to be a completely inorganic optical material that can perform even under high-temperature conditions. In fact, the noncombustibility of BoL-LC was demonstrated by exposure to direct fire. This property was totally different from that of graphene or other organic materials (Supplementary Movie 3). In addition, BoL-LC and BoL-C have sufficient solubility in organic solvents (Supplementary Movie 4). These fundamental features highlight the advantages of these materials in a wide range of applications. To examine the feasibility of such use, we assessed the electronic conductivity of BoL-LC in the P-i phase by direct-current current–voltage (*I*–*V*) measurements (Supplementary Fig. 11). The increase in current in BoL-LC at applied voltages greater than 1 V indicates that it could have applications in on/off optical switching based on dynamic scattering (Fig. 4a)^33^. Upon applying BoL-LC to a comb electrode (Fig. 4b), it was found to move dynamically in response to an applied voltage (Supplementary Movie 5). A reversible on/off-type transition of transmitted light was observed when using only 1 V with an alternating current (Fig. 4c). The applied voltage for dynamic scattering was extraordinarily low compared with that in the case of common organic LC devices^34^. The observed response times of ~25–60 ms were slower than those of a representative device fabricated using anisylidene-*p*-aminophenylacetate (APAPA) but were comparable to those of various organic LC devices (Fig. 4d, e, Supplementary Table 1)^16,35^.

The demonstrated on/off device made of BoL-LC was based on a dynamic scattering mode (DSM), which has been used to investigate display applications. However, further development is hindered by the high driving voltage. This mode often occurs when the preferred directions of dielectric properties and conductivity are different^36^. In the case of BoL-LC, single-layered cations (K^+^) and anionic layers (BoLs) are laminated. Therefore, a high dielectric constant is expected along the stacking direction. However, a nearly zero activation energy in the in-plane direction of BoL has been reported^9^. These reasonable structures cause a lower driving voltage of devices generating DSM than common materials. Furthermore, the many potassium cations in BoL-LC also play a role in inducing dynamic scattering by active movement at the application of voltage. The demonstrated behavior is derived from the dynamics of droplets. Therefore, further detailed evaluation requires a uniform thickness for the preparation of devices. The advantage of the inorganic nature of BoLs was confirmed by their stability. This on/off switching device was active under a wide thermal range from 130 to 280 °C (Fig. 4f). Considering the noncombustible characteristics of the device, these results demonstrate the feasibility of inorganic liquid devices that can be used in harsh environments. In addition, the range can be expanded by a simple chemical modification. The dehydration reaction from BoL-C with CH_3_CN and EtOH (1 wt%) gave a BoL-LC droplet, which is a P-i phase, even around room temperature (Supplementary Fig. 12). We note that almost all of the solvents (CH_3_CN and EtOH) evaporated by heating at 200 °C under vacuum conditions. Therefore, we consider that chemical modification by EtOH molecules triggered this phenomenon.

## Discussion

We have investigated a liquid inorganic material derived from a borophene analogue. The material was generated by a dehydration reaction at the edge part of BoL-C, which weakened the interactions between interlayers. The structure and reaction were revealed by optical or electron microscopy and DSC, TG, IR and XPS measurements. The liquid phase of BoL-LC with an ordered layer structure can exist over an extraordinarily wide temperature range. The demonstrated dynamic scattering device was driven by a low voltage of 1 V. We also observed the highly ordered orientation of P-ii. This orientation is expected to be useful for uniform coatings of BoL-LC on substrates. The results of the present work indicate that BoL-LC exhibits strong potential for use in widespread applications that are unavailable to conventional organic liquid crystals or inorganic materials.

## Methods

### Materials and characterization

BoL-C was prepared by dissolving KBH_4_ in CH_3_CN solution via a partial oxidation reaction according to the previously reported method^9^. The obtained colorless BoL-C crytstals were confirmed by powder XRD measurements. Polarized optical microscopy images were acquired using an Olympus BX50, BX53-P and a Nikon Eclipse E400 POL with halogen lamps controlling the temperature of specimen with a METTLER TOLEDO FP-90 or FP-82HT. Figure 1d was observed with a sensitive tint plate. Berek compensator (Olympus U-CBE), a color filter (green, 45-IF546, Olympus) and a tint plate (U-TP530, Olympus) were used for retardation measurements. During the measurements, temperatures were controlled using HCS402 (INSTEC). The samples for optical measurement shown in Supplementary Figs. 9 and 10 were prepared using glass cells and polyimide film (Kapton). For the BoL-C sample, BoL-C was placed on a glass plate (1.3 mm) surrounded with polyimide film as a spacer, and another glass plate (1.3 mm) was put on the top of them to create a cell. The sides were fastened with Araldite bond (ARALDITE RT30). For the BoL-LC sample, BoL-C between two glass plates was heated at 200 °C for 2 h under vacuum condition to change to BoL-LC. After cooling to room temperature, four sides of the cell were sealed in the same way. DSC curves were recorded using a DSC 8230 (Rigaku) and DSC 7000X (Hitachi High-Tech Science Corporation). Mark-tubes (0.7 mm, Hilgenberg) were used as glass capillaries. TGA curves were acquired via a TG 8120 (Rigaku) at a heating rate of 5 °C/min. TG-MS spectra were obtained using a ThermoMass Photo (Rigaku) at a heating rate of 5 °C/min. FT-IR spectra were obtained using a JASCO FT/IR-4700; samples were prepared with KBr pellets. Field-emission SEM observations were carried out with an S5500 instrument (HITACHI High Technology) operating at 5 kV. XPS was performed using a PHI 5000 VersaProbe (Ulvac-Phi, Inc.) equipped with an Al K*α* (15 kV, 25 W) radiation source focused on a 100 μm^2^ sample area; spectra were analyzed using the MultiPak software package (Physical Electronics). Spectra were standardized on the basis of the C 1 *s* peak at 284.6 eV. TEM images were obtained using a JEOL JEM-ARM200F; samples were deposited onto a Cu mesh microgrid (Okenshoji Co.). Electronic conductivity measurements were performed via the impedance method in conjunction with a BAS ALS750B analyzer and conductive electrodes purchased from BAS, Inc. An optical device cell with comb electrodes (indium-doped tin oxide (ITO) electrodes, distance between electrodes: 5 µm, electrode width: 10 μm) was also obtained from BAS, Inc. Voltage was applied using HSA4011 and WF1973 units (NF Corp.). The BoL-LC device was prepared by heating BoL-C on the comb electrodes; device observations were carried out using polarized optical microscopy.

## Supplementary information

Supplementary Information

Description of Additional Supplementary Files

Supplementary Movie 1

Supplementary Movie 2

Supplementary Movie 3

Supplementary Movie 4

Supplementary Movie 5

## Data Availability

All data generated and analyzed during this study are included in this article and its Supplementary Information, and are also available from the authors upon reasonable request.
