# Supplementary material for: Liquid crystalline 2D borophene oxide for inorganic optical devices
Source: Nat Commun. 2022 Feb 24;13:1037. doi: 10.1038/s41467-022-28625-w (PMC8873452; doi:10.1038/s41467-022-28625-w)
Supplement: Supplementary file 1 — Supplementary Information [file 41467_2022_28625_MOESM1_ESM.pdf]

Supplementary Information

Liquid crystalline 2D borophene oxide for inorganic optical devices

Authors

Tetsuya Kambe[1,2], Shotaro Imaoka[1], Misa Shimizu[1], Reina Hosono[1], Dongwan Yan[3], Hinayo Taya[1], Masahiro Katakura[1], Hirona Nakamura[1], Shoichi Kubo[1], Atsushi Shishido[1], Kimihisa Yamamoto[1,2]*

Affiliations

[1] Laboratory for Chemistry and Life Science, Tokyo Institute of Technology, Yokohama 226-8503, Japan

[2] JST-ERATO, Tokyo Institute of Technology, Yokohama 226-8503, Japan

[3] Kanagawa Institute of Industrial Science and Technology (KISTEC), Tokyo Institute of Technology, Yokohama 226-8503, Japan

*Correspondence to: yamamoto@res.titech.ac.jp

This file includes:

Supplementary Figures 1 to 12
Supplementary Table 1

Other Supplementary Materials for this manuscript includes the following:

Supplementary Movies 1 to 5

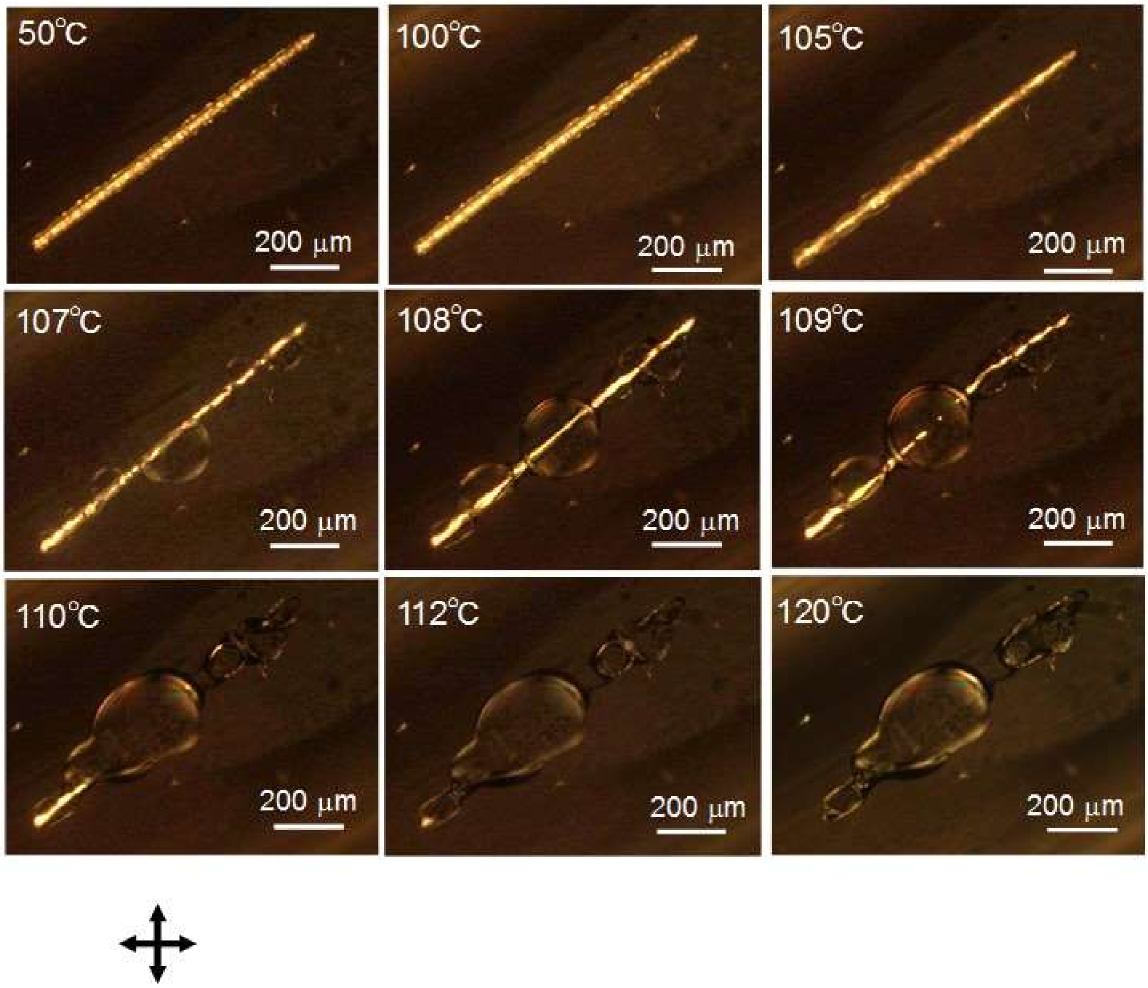

Supplementary Figure 1

Change from BoL-C to BoL-LC. The photographs are measured by polarized optical microscopy under crossed polarizers (black double arrows) during heating process.

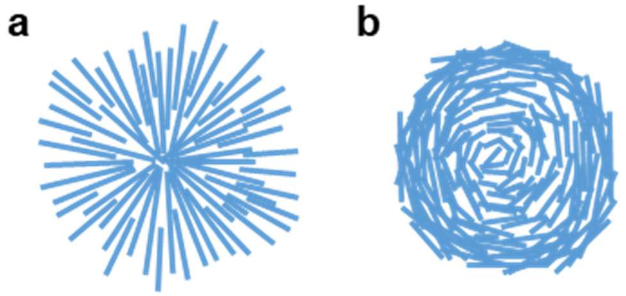

Supplementary Figure 2

Models of spherulite structures. a, b, Schematic illustrations show possible conformation of the spherulite structure *via* the arrangement of two-dimensional sheets. The blue bars show a side view of the sheets from the spherulite.

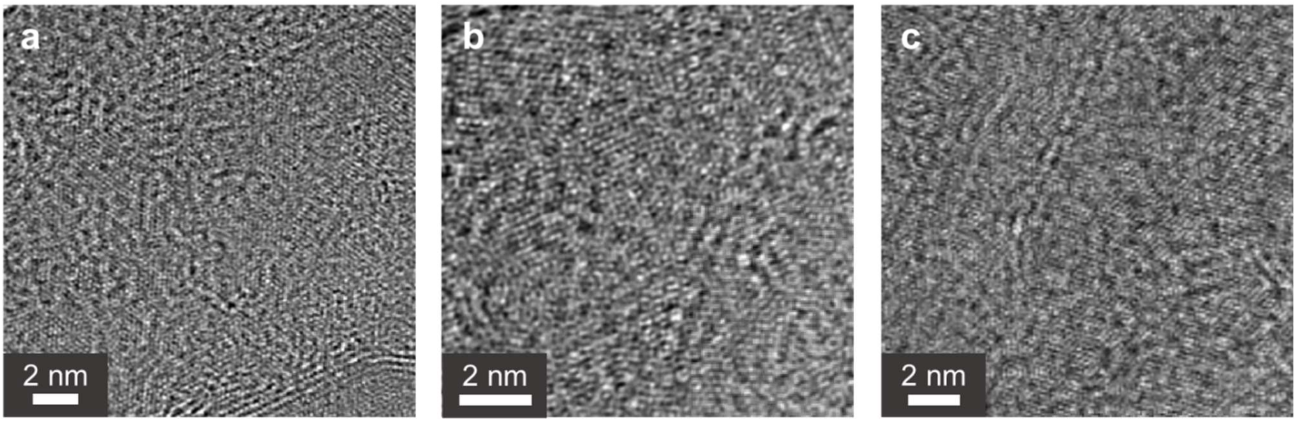

Supplementary Figure 3

TEM images of BoL-LC. a-c, Closed-up TEM images corresponding to single (**a**), double (**b**), and several layers (**c**), respectively. These layers were confirmed by selected-area electron diffraction.

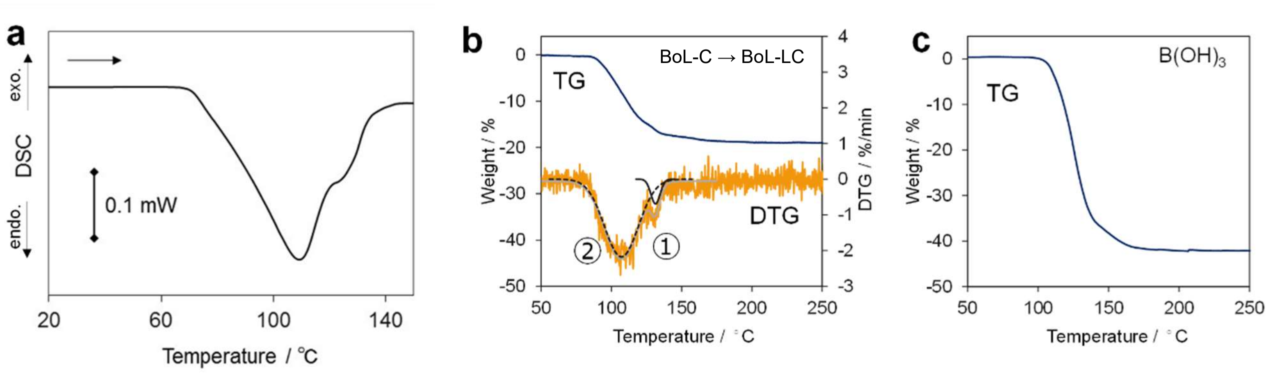

Supplementary Figure 4

TG and DSC curves for the change from BoL-C to BoL-LC. a, A DSC curve of the BoL-C during a heating process under inert gas condition. **b,** Thermogravimetric and differential thermogravimetric curves of the BoL-C during the heating process. Peak 1 corresponds to the removal of H_2O from the terminal B-OH units. **c,** A thermogravimetric curve of $B(OH)_3$. The temperature of the peak 1 in (**b**) matched that of the weight-decreasing in (**c**).

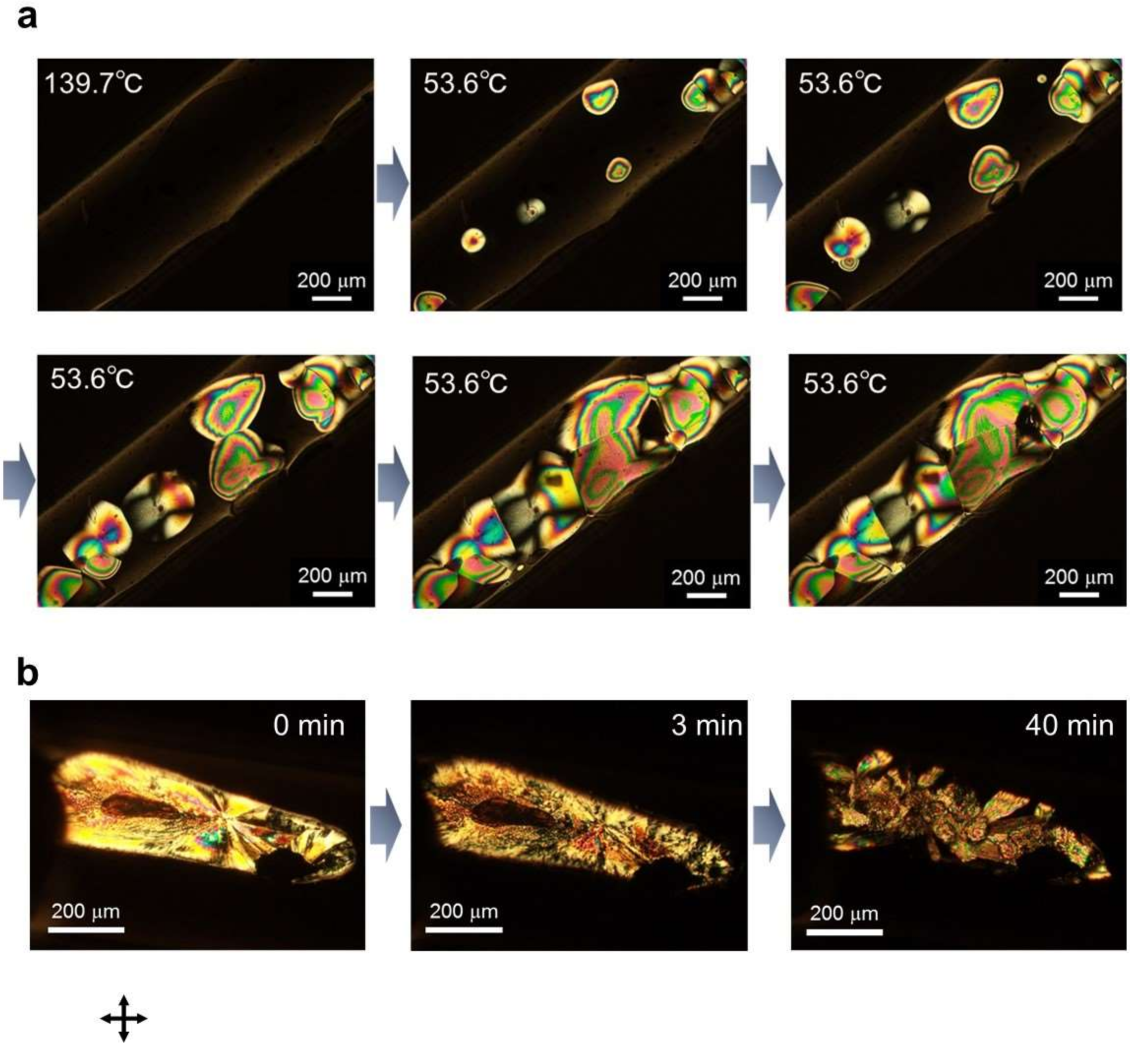

Supplementary Figure 5

Polarized optical microscopy images. a, The phase change from P-i to P-ii. The images were captured by a polarized microscopy. The measurement conditions including brightness were adjusted to P-ii. **b,** Changes from crystal to P-ii phase. The photographs show time-dependent polarized optical microscopic images after rapid-cooling from 200 °C (P-i) to room temperature. Waiting times are 0, 3 and 40 min. The radial straight lines on the sample (0 min) suggested the crystalline phase, and they gradually disappeared.

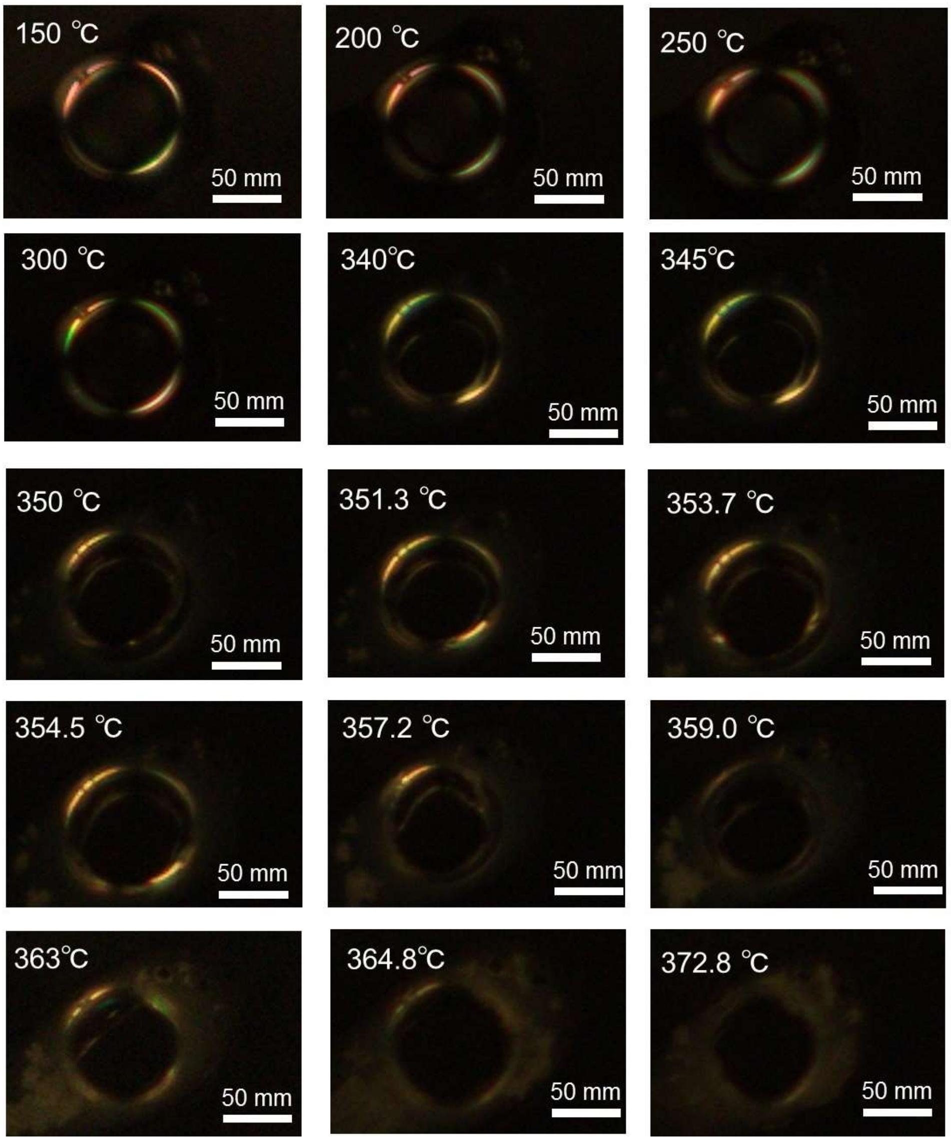

Supplementary Figure 6

Polarized optical microscopic images under crossed polarizers during heating process. The bright periphery with four dark points at a 90-degree separated position indicates the liquid crystalline phase. The LC phase was kept until 350 °C. It began to blink from 355 °C, and completely disappeared over 370 °C.

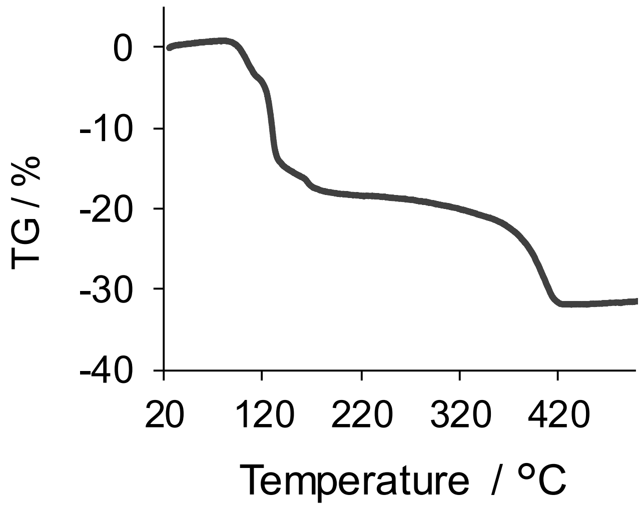

Supplementary Figure 7

Thermogravimetric curve of BoL-C. BoL-C changed to BoL-LC after dehydration reaction around 120 °C. Then BoL-LC thermally decomposed with decreasing of the weight from 370 °C.

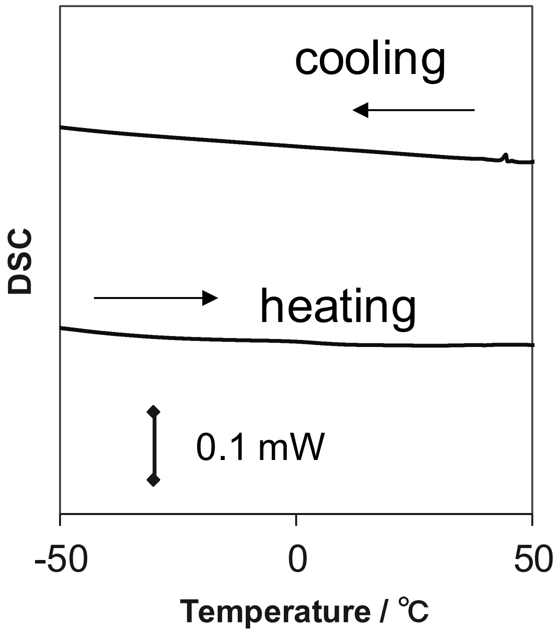

Supplementary Figure 8
DSC curves of BoL-LC at low temperature.

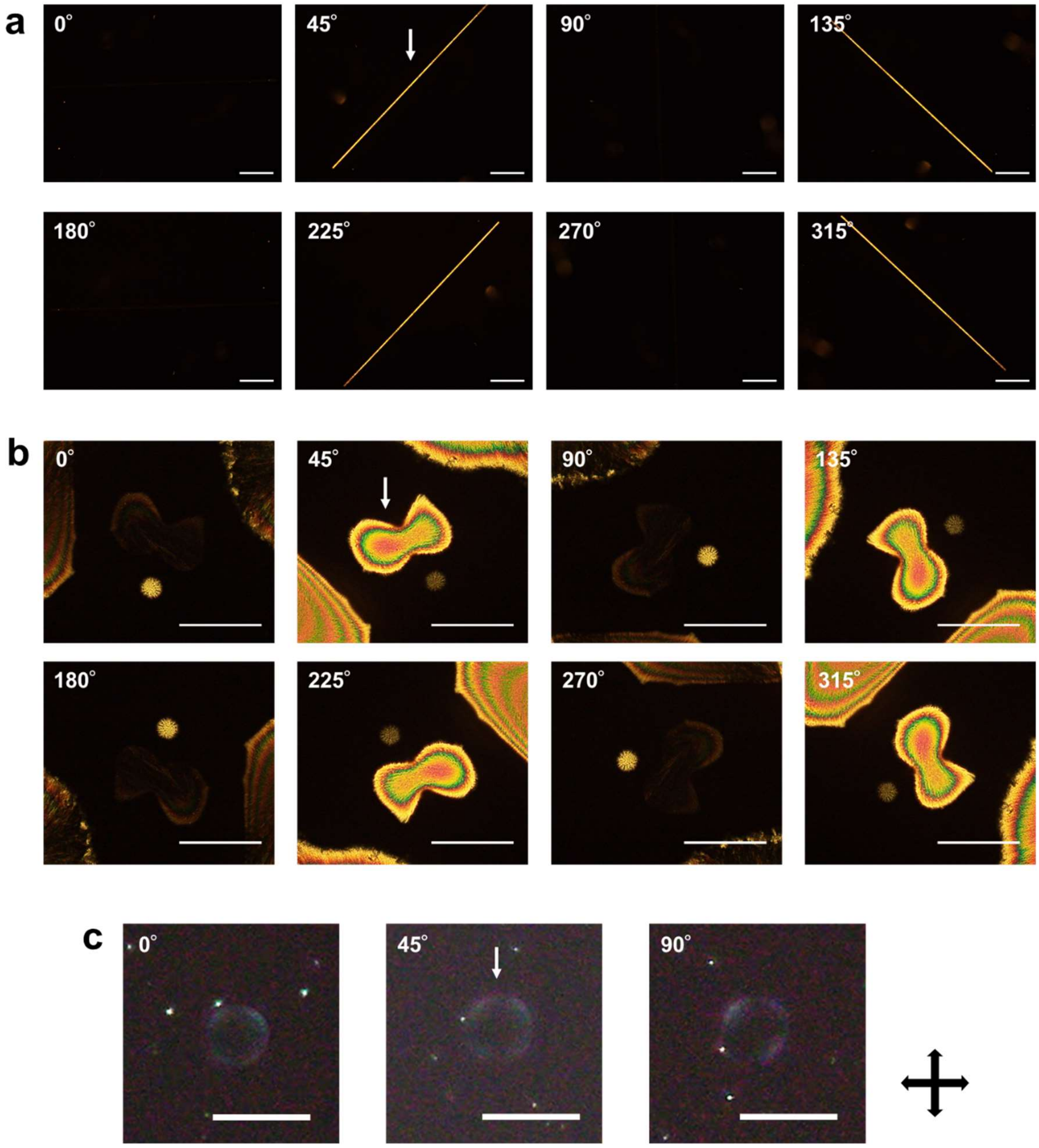

Supplementary Figure 9

Optical microscopic images of a) BoL-C, b) BoL-LC (P-ii) and c) BoL-CL (P-i). Directions of polarizer and analyzer are represented as two black crossed arrows (right down). The white scale bars mean 500 μm (**a**), 200 μm (**b**) and 50 μm (**c**). The degrees shown at top left in each photograph mean relative angles of the measured sample. White arrows in the photographs of 45° indicate the position of representative samples.

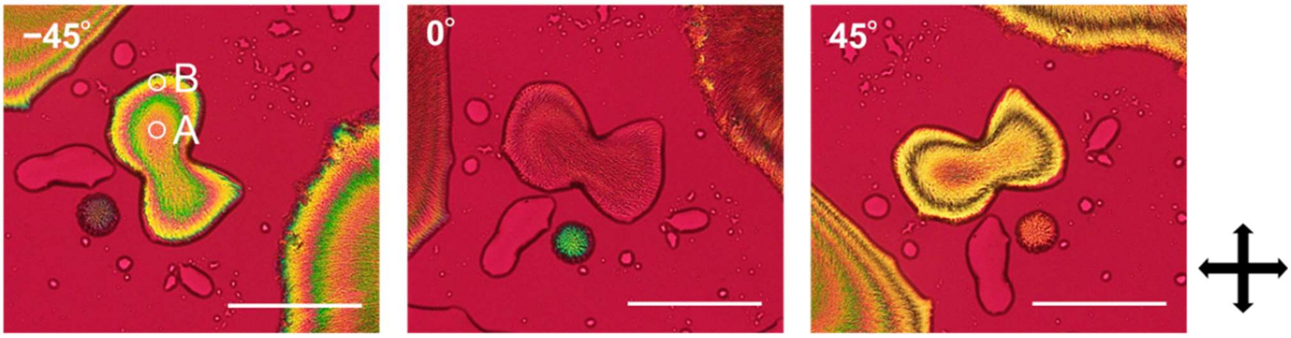

Supplementary Figure 10

Optical microscopic images of BoL-LC (P-ii) with a tint plate. Directions of polarizer and analyzer are represented with black crossed arrows. The scale bars mean 200 μm. The degrees shown at top left in each photograph mean relative angles of the measured sample. The retardation (*R*) and birefringence (Δn) values were estimated at the positions of A and B.

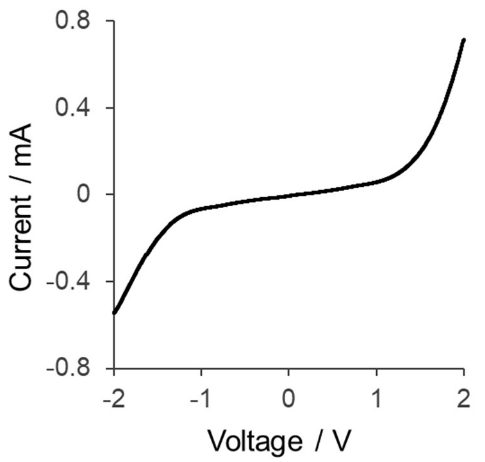

Supplementary Figure 11
I-V curve of the BoL-LC on a comb electrode.

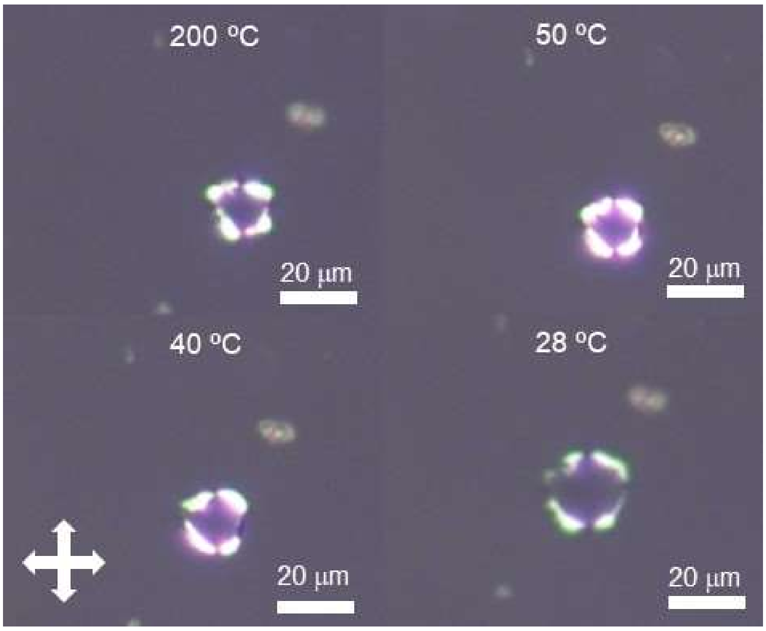

Supplementary Figure 12

Optical microscopic images of modified BoL-LC droplet. The BoL-C was treated with EtOH (1 wt% in CH_3CN) before dehydration reaction at 200 °C under a vacuumed condition.

Supplementary Table 1. Decay and rise times of LCs.

	Decay time / msec.	Rise time / msec.
BoL-LC	~25	~50
APAPA	1-5	-30
General dynamic scattering type LC	10-50	30-150
