# Supplementary material for: Liquid crystalline 2D borophene oxide for inorganic optical devices
Source: Nat Commun. 2022 Feb 24;13:1037. doi: 10.1038/s41467-022-28625-w (PMC8873452; doi:10.1038/s41467-022-28625-w)
Supplement: Supplementary file 2 — Description of Additional Supplementary Files [file 41467_2022_28625_MOESM2_ESM.pdf]

Description of Additional Supplementary Files

Supplementary Movie 1: Generation of BoL-LC from BoL-C. This microscope movie shows how BoL-C changes to BoL-LC through the dehydration reaction.

Supplementary Movie 2: Self-organization ability of BoL-LC. One spherulite droplet of BoL-LC is transformed into three spherulite droplets by division. This feature is compared with the solid BoL-C.

Supplementary Movie 3: Incombustibility of BoLLC. BoL-LC and graphene nanoplatelets on quartz substrates are exposed to direct fire. Unlike graphene derivatives, BoL-LC is incombustible even at the decomposed temperature over 370 ºC.

Supplementary Movie 4: Dissolution behavior of BoL-C. BoL-C (left) or graphene nanoplatelets (right) are added to the N-methylpyrrolidone at 150 ºC. In this temperature, BoL-C can change to BoL-LC in the solvent by the dehydration reaction. The BoL sample dissolves rapidly, while graphene nanoplatelets do not.

Supplementary Movie 5: Dynamic moving of BoLLC on electrodes. The liquid BoL-LC is placed sparsely on a comb electrode (dark lines from top right to bottom left). BoL-LC, which consists of ordered 2D atomic sheets, exhibits dynamic activity under an alternating voltage (5 V, 1 Hz) at 50 ºC.
